# Exploration of first onsets of mania, schizophrenia spectrum disorders and major depressive disorder in perimenopause

**DOI:** 10.1038/s44220-024-00292-4

**Published:** 2024-08-15

**Authors:** Lisa M. Shitomi-Jones, Clare Dolman, Ian Jones, George Kirov, Valentina Escott-Price, Sophie E. Legge, Arianna Di Florio

**Affiliations:** 1 Centre for Neuropsychiatric Genetics and Genomics, Division of Psychological Medicine and Clinical Neurosciences, School of Medicine, Cardiff University, Cardiff, UK; 2 Bipolar UK, London, UK; 3 National Centre for Mental Health, School of Medicine, Cardiff University, Cardiff, UK

**Keywords:** Bipolar disorder, Psychosis, Schizophrenia, Depression

## Abstract

Although the relationship between perimenopause and changes in mood has been well established, knowledge of risk of a broad spectrum of psychiatric disorders associated with reproductive aging is limited. Here we investigate whether the perimenopause (that is, the years around the final menstrual period (FMP)) is associated with increased risk of developing psychiatric disorders compared with the late reproductive stage. Information on menopausal timing and psychiatric history was obtained from nurse-administered interviews and online questionnaires from 128,294 female participants within UK Biobank. Incidence rates of psychiatric disorders during the perimenopause (4 years surrounding the FMP) were compared with the reference premenopausal period (6–10 years before the FMP). The rates were calculated for major depressive disorder (MDD), mania, schizophrenia spectrum disorders and other diagnoses. Overall, of 128,294 participants, 753 (0.59%) reported their first onset of a psychiatric disorder during the late reproductive stage (incidence rate 1.53 per 1,000 person-years) and 1,133 (0.88%) during the perimenopause (incidence rate 2.33 per 1,000 person-years). Compared with the reference reproductive period, incidence rates of psychiatric disorders significantly increased during the perimenopause (incidence rate ratio (RR) of 1.52, 95% confidence interval (CI) 1.39–1.67) and decreased back down to that observed in the premenopausal period in the postmenopause (RR of 1.09 (95% CI 0.98–1.21)). The effect was primarily driven by increased incidence rates of MDD, with an incidence RR of 1.30 (95% CI 1.16–1.45). However, the largest effect size at perimenopause was observed for mania (RR of 2.12 (95% CI 1.30–3.52)). No association was found between perimenopause and incidence rates of schizophrenia spectrum disorders (RR of 0.95 (95% CI 0.48–1.88)). In conclusion, perimenopause was associated with an increased risk of developing MDD and mania. No association was found between perimenopause and first onsets of schizophrenia spectrum disorders.

## Abstract (web summary)

The authors investigate first onsets of psychiatric disorders during perimenopause, finding higher incidence rates of major depressive disorder and mania.

## Main

There are estimated to be greater than 945 million women and those assigned female at birth aged between 40 and 60 years in the world^1^. During perimenopause (the years around the final menstrual period (FMP)), approximately 80% of people develop neuropsychiatric symptoms, most commonly hot flushes, cognitive dysfunction, sleep disturbances and mood-related symptoms^2^. It has been suggested that perimenopause is also a high-risk period for the onset or exacerbation of psychiatric disorders, including major depressive disorder (MDD), schizophrenia spectrum disorders and bipolar disorder; although, research thus far has predominately measured only depressive symptoms^3–7^. A two- to four-times greater risk of a depressive episode during perimenopause compared with the reproductive stage has been observed by the limited studies that have investigated MDD, even after controlling for other predictors and perimenopausal symptoms^8–10^. However, failure to account for multiple testing, retrospective design and focus on mild symptomatology have limited the generalizability of the results. Very little research has been conducted on the association between perimenopause and onset of disorders such as schizophrenia and bipolar disorder. Observations thus far have been limited to risk of recurrence in women with preexisting disorders and have shown that midlife in women is associated with worsening bipolar symptoms^11^, as well as an increased risk of hospitalizations for psychosis compared with men of the same age^12^. A major limitation of researching the association between reproductive aging and psychiatric disorders is related to the difficulties in obtaining reliable evaluations of ovarian aging, especially in epidemiological studies, with most studies using age as a proxy for age at FMP^13^. However, there is an over 20-year range variation in age at menopause, with research advocating that chronological age is not a valid proxy for menopausal status^14–16^. The UK Biobank represents a unique opportunity to study the effects of reproductive aging, with information on menopausal timing and deep longitudinal data collected on over 200,000 female participants recruited at 40–69 years of age^17^. Here, we focus on ‘late’ first onsets, because studies on the effect of reproductive aging on first-onset severe mental illness (that is, schizophrenia spectrum disorders and bipolar disorder) are lacking. This is likely due to the low prevalence and, therefore, difficulty to detect any effect in epidemiological studies specifically designed to study menopause. In this article, we aim to exploit a unique and specific asset of the UK Biobank: the combination of questions on first-onset psychiatric illness and age at FMP in an epidemiological study large enough to detect less prevalent, devastating outcomes, such as first incidence of bipolar disorder/schizophrenia, at a population level. The aim of this study is to test the hypothesis that perimenopause is a time of increased rate of first-onset psychiatric disorders, including MDD, mania and schizophrenia spectrum disorders, compared with the late reproductive stage.

## Results

**Fig. 1:**
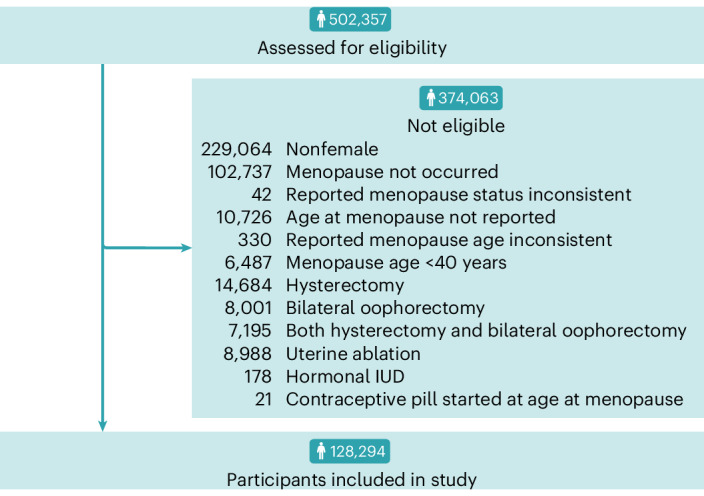
Flowchart of participant selection criteria and quality control. Please note that each exclusion criterion was implemented sequentially, with the number of participants removed at each stage calculated on the basis of the participants remaining after the previous criteria had been applied.

**Fig. 2:**
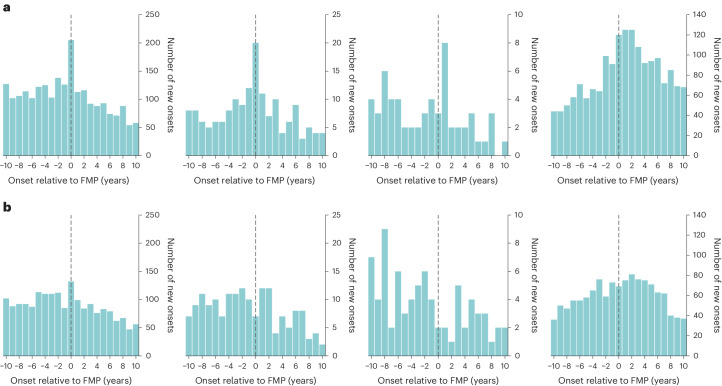
Age of first onset of psychiatric disorders relative to age at FMP. **a**, Female participants for MDD (left), mania (middle left), schizophrenia spectrum disorders (middle right) and other diagnoses (right). **b**, Male participants for MDD (left), mania (middle left), schizophrenia spectrum disorders (middle right) and other diagnoses (right). Please note that not all participants were followed up for 10 years after the FMP (or matched ‘FMP’ proxy for male participants), and thus, later numbers of new onsets are likely to be underestimated.

A total of 128,294 participants from the UK Biobank met inclusion criteria, as shown in Fig. 1. The mean age at recruitment into this study was 59.6 years (standard deviation (s.d.) of 5.69 years) with an average follow-up period of 2.98 years (s.d. of 3.92 years) and mean age at menopause of 50.6 years of age (range of 40–68 years; s.d. of 4.00 years), as displayed in Supplementary Fig. 1. Demographic characteristics of female participants, including self-reported ethnicity, are presented in Table 1. As presented in Table 2, a total of 753 participants (0.59%) had a first onset of a psychiatric disorder 6–10 years before the FMP (premenopause period), 1,133 participants (0.88%) between 2 years prior and 2 years following FMP (perimenopause) and 637 participants (0.50%) 6–10 years following the FMP (postmenopause), with incidence rates per 1,000 person-years of 1.53, 2.33 and 1.66, respectively. Trends in the number of new onsets of each psychiatric disorder for both female and male participants are displayed in Fig. 2.

**Table 1:** Demographic characteristics

Demographic variable	*N*	%	Mean	s.d.
Age at first assessment (years)			59.6	5.69
Age at most recent follow-up (years)			62.5	6.30
Age at FMP (years)			50.6	4.00
Ethnicity				
White^a^	122,652	95.60		
Black or Black British^b^	1,393	1.09		
Mixed^c^	600	0.47		
Indian	1,297	1.01		
Pakistani	225	0.18		
Bangladeshi	22	0.02		
Chinese	405	0.32		
Other Asian background^d^	351	0.27		
Other ethnic group^d^	1,013	0.79		
Do not know/prefer not to say	336	0.26		

Ethnic groups have been organized to reflect the same categories as Fry et al.^30^, who in turn used these categories to allow for comparison with the 2001 and 2011 UK Census data.

^a^Includes white British, white Irish and any other white background.

^b^Includes Black Caribbean, Black African and any other Black background.

^c^Includes white and Black Caribbean, white and Black African, white and Asian and any other mixed background.

^d^‘Other Asian background’ and ‘Other ethnic group’ are both specific responses available from the question posed by the UK Biobank and are not further broken down into more granular categories.

**Table 2:** Incidence rates of psychiatric disorders during the perimenopause and postmenopause compared with the premenopause stage

Disorder	Life stage	*N*	New onsets	Rate per 1,000 person-years	RR (95% CI)	*P*	Adjusted *P*
Psychiatric disorder	Premenopause	123,120	753	1.53	1.00 (reference)		
	Perimenopause	121,764	1,133	2.33	1.52 (1.39–1.67)*	2.34 × 10^−19^*	3.89 × 10^−18^*
	Postmenopause	95,690	637	1.66	1.09 (0.98–1.21)	1.22 × 10^−1^	1.91 × 10^−1^
Major depressive disorder	Premenopause	39,800	551	3.46	1.00 (reference)		
	Perimenopause	38,899	698	4.49	1.30 (1.16–1.45)*	5.63 × 10^−6^*	2.56 × 10^−5^*
	Postmenopause	36,642	345	2.35	0.68 (0.59–0.78)*	1.63 × 10^−8^*	1.16 × 10^−7^*
Mania	Premenopause	128,105	26	0.05	1.00 (reference)		
	Perimenopause	128,051	55	0.11	2.12 (1.30–3.52)*	1.68 × 10^−3^*	4.42 × 10^−3^*
	Postmenopause	90,300	18	0.05	0.98 (0.51–1.86)	1.00	1.00
Schizophrenia spectrum disorders	Premenopause	128,170	20	0.04	1.00 (reference)		
	Perimenopause	128,144	19	0.04	0.95 (0.48–1.88)	1.00	1.00
	Postmenopause	102,879	6	0.01	0.37 (0.12–0.96)*	4.05 × 10^−2^*	7.79 × 10^−2^
Other diagnoses	Premenopause	127,292	211	0.41	1.00 (reference)		
	Perimenopause	126,798	442	0.87	2.10 (1.78–2.49)*	5.91 × 10^−20^*	1.48 × 10^−18^*
	Postmenopause	89,053	307	0.86	2.08 (1.74–2.49)*	1.70 × 10^−16^*	2.13 × 10^−15^*

This table displays the incidence RRs associated with each psychiatric disorder calculated from comparisons between different life stages. The first column lists psychiatric disorders. Incidence rates are calculated as the number of first onsets per 1,000 person-years. Incidence RRs are calculated as the ratio associated with each life stage, relative to the reference reproductive stage.

*Significant at *α* = 0.05 confidence level; reproductive is 6–10 years before FMP, perimenopause is between 2 years before and 2 years after FMP and postmenopause is 6–10 years after FMP.

Adjusted *P* values represent the false discovery rate-adjusted *P* values, accounting for all tests conducted in Table 2 and Supplementary Tables 1, 3 and 4.

The perimenopause was associated with a significant increase in the incidence rates of psychiatric disorders compared with the premenopause period (incidence rate ratio (RR) of 1.52 (95% confidence interval (CI) 1.39–1.67); Table 2). During the postmenopause, the incidence rate decreased back down to that observed in the premenopause period (RR of 1.09 (95% CI 0.98–1.21)).

### Disorder-specific analyses

**Fig. 3:**
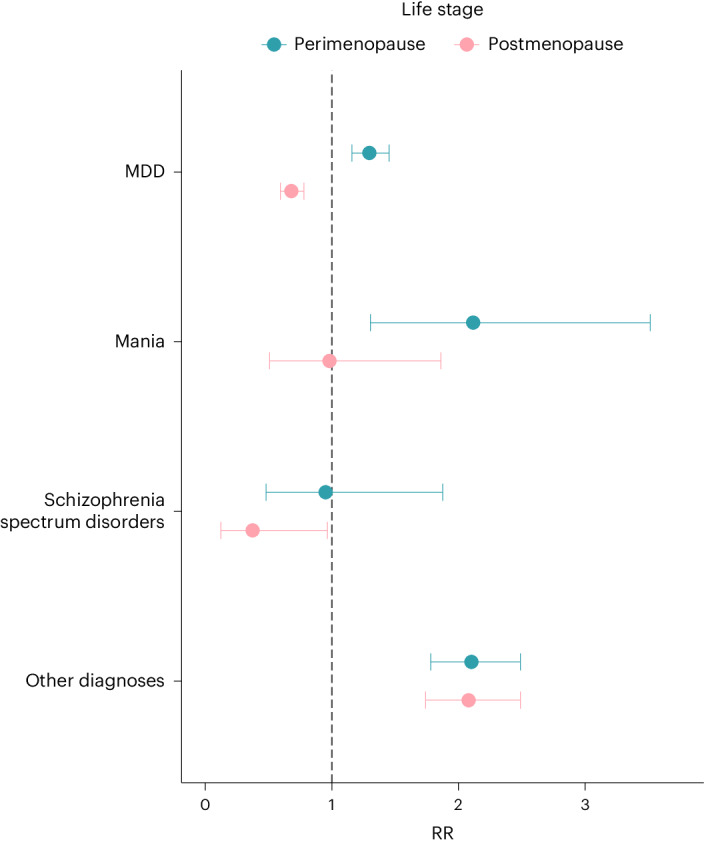
Incidence RRs of first onset of psychiatric disorders during perimenopause and postmenopause, relative to the premenopause. The dots indicate the incidence RR calculated relative to the premenopausal period (6–10 years before FMP). The whiskers indicate 95% CIs. Total sample sizes analyzed for each disorder are as follows: MDD (*n* = 39,800), mania (*n* = 128,105), schizophrenia spectrum disorders (*n* = 128,170) and other diagnoses (*n* = 127,292). Perimenopause is between 2 years before and 2 years after FMP, and postmenopause is 6–10 years after FMP.

MDD was found to have a higher incidence rate during the perimenopause compared with the premenopause stage, with a RR of 1.30 (95% CI 1.16–1.45) (Fig. 3 and Table 2). The incidence rate of MDD became significantly lower in the postmenopause compared with the premenopause (RR of 0.68 (95% CI 0.59–0.78)).

The incidence rate of mania significantly increased in the perimenopause compared with the premenopause (RR of 2.12 (95% CI 1.30–3.52)), to then return to the premenopause level during the postmenopause (RR of 0.98 (95% CI 0.51–1.86)).

Perimenopause was not found to be significantly associated with a change in incidence rate of schizophrenia spectrum disorders compared with the premenopause stage (RR of 0.95 (95% CI 0.48–1.88)), though rates were found to be lower during the postmenopause (RR of 0.37 (95% CI 0.12–0.96)).

Both perimenopause and postmenopause were found to be significantly associated with an increased incidence rate of other diagnoses compared with the premenopause, with incidence RRs of 2.10 (95% CI 1.78–2.49) and 2.08 (95% CI 1.74–2.49), respectively.

### Sensitivity analysis

Sensitivity analyses did not detect any effect of potential confounders on results. Compared with the premenopause, an association between perimenopause and an increased incidence rate of psychiatric disorders was observed in those with low and high Townsend Deprivation Indexes, those of healthy, preobese and obese body mass index (BMI) categories, across all six alcohol intake frequency categories and in both previous and never smokers (Supplementary Table 1). Perimenopause was not found to be significantly associated with an increase in incidence rate of psychiatric disorders compared with the premenopause in those with underweight BMI or current smokers.

### Analysis of male participants

**Extended Data Fig. 1:**
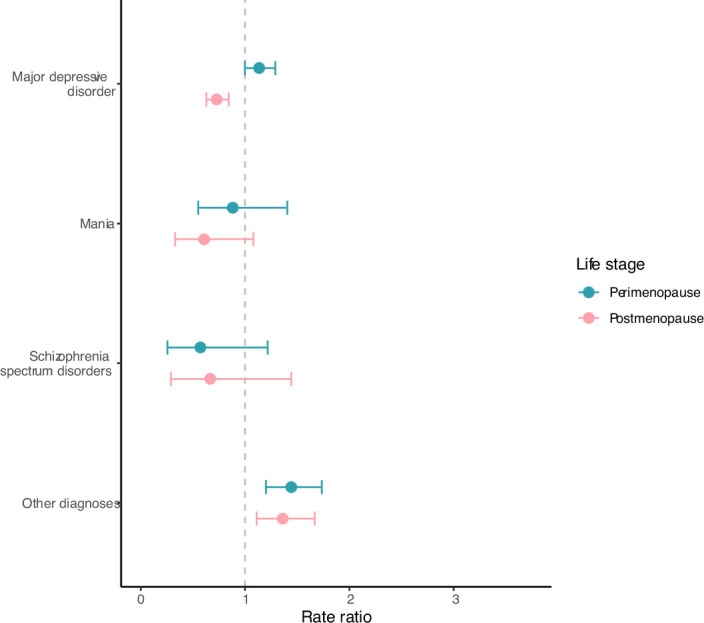
Incidence rate ratios of psychiatric disorders for male participants during matched ‘perimenopause’ and ‘postmenopause’ proxies, relative to the matched ‘premenopause’ period proxy. Note that age-based classifications were based on values from matched female participants. Dots indicate the incidence rate ratio calculated relative to the premenopausal period proxy (6-10 years before the assigned FMP). Whiskers indicate 95% confidence intervals. Total sample sizes analysed for each disorder are as follows: major depressive disorder (n = 37 719); mania (n = 128 131), schizophrenia spectrum disorders (n = 128 108); other diagnoses (n = 127 449). Perimenopause = Between −2 years before and 2 years after FMP; Postmenopause = 6-10 years after FMP.

**Extended Data Fig. 2:**
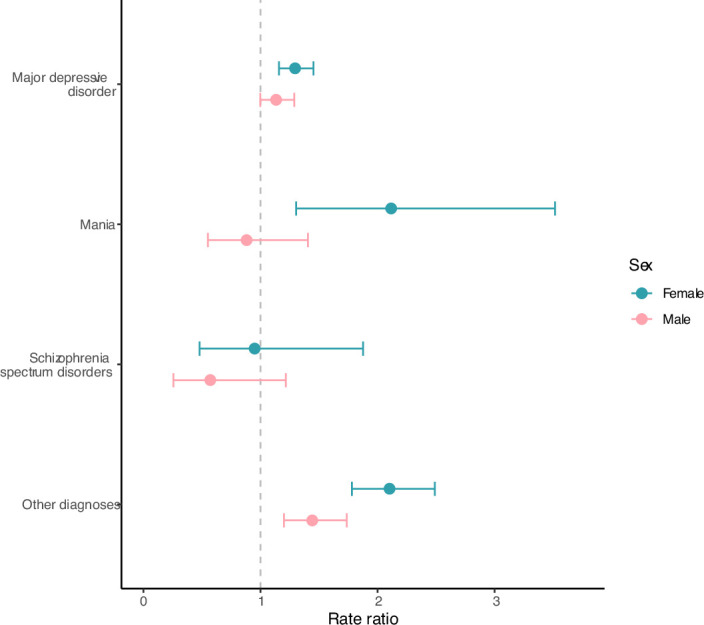
Incidence rate ratios of psychiatric disorders in female participants during the perimenopause (2 years prior - 2 years following FMP) compared to male participants during the matched ‘perimenopause’ proxy (2 years prior - 2 years following assigned FMP). Note that age at FMP in male participants was based on values from matched female participants. Dots indicate the incidence rate ratio calculated relative to the premenopausal period (6-10 years before FMP). Whiskers indicate 95% confidence intervals. Sample sizes analysed for each disorder are as follows: major depressive disorder (n_female_ = 39 800, n_male_ = 37 719); mania (n_female_ = 128 105, n_male_ = 128 131), schizophrenia spectrum disorders (n_female_ = 128 170, n_male_ = 128 108); other diagnoses (n_female_ = 127 292, n_male_ = 127 449). Perimenopause = Between −2 years before and 2 years after FMP; Postmenopause = 6-10 years after FMP.

Demographic characteristics of male participants are available in Supplementary Table 2. No individual disorder had any significant peak of incidence at the interval period that matched ‘perimenopause’ (Extended Data Fig. 1). First onsets of MDD had a similar pattern in male and female participants; although, this did not reach statistical significance in the male sample during the ‘perimenopause’ proxy (RR of 1.13 (95% CI 1.00–1.29); Extended Data Fig. 2 and Supplementary Table 3). In male participants, no increase in incidence rate was observed during the ‘perimenopause’ proxy compared with the ‘premenopause’ proxy for mania (RR of 0.88 (95% CI 0.55–1.41)) nor schizophrenia spectrum disorders (RR of 0.57 (95% CI 0.26–1.22); Supplementary Table 3). Similarly to the female sample, both the perimenopause and postmenopause proxies in male participants were associated with an increased rate of other diagnoses compared with the premenopause proxy, with incidence RRs of 1.44 (95% CI 1.20–1.74) and 1.36 (95% CI 1.11–1.67), respectively.

## Discussion

Our results show that the perimenopause is a period of increased risk of first-onset psychiatric disorders, with compelling evidence for a specific link with mania. We found that participants without previous history of mania were over twice as likely to develop mania for the first time in the perimenopause than in the late reproductive stage. The increased risk was specific for the perimenopause, as the rates of first-onset mania returned to premenopause levels in the postmenopause.

### Main findings

Our results highlight the importance of considering the FMP rather than chronological age in both clinical practice and research concerning mental health and reproductive aging. Previous studies using chronological age had not been able to detect the effect of perimenopause on mania/bipolar disorder^18^. Given the 20-year range variation in age at FMP^14^, inferring age at menopause on solely chronological age can lead to errors and, in research, to false negatives. The disease-specific and narrow time window (4 years) of increased risk for mania also suggests that specific changes associated with the perimenopause may trigger mania in people without previous psychiatric history of mania. The lack of any association in male participants corroborates the idea that sex-specific factors are associated with the first occurrence of mania at the time of the perimenopause. For bipolar disorder and mania, evidence suggests a trimodal distribution of age of onset, reflecting possible biological heterogeneity^19^. By selecting a period of 4–10 years prior the FMP as reference, our analyses focused on the late onset group, providing for the first time direct evidence of a possible link between mania and the hormonal fluctuations of the perimenopause. Intriguingly, one of the most robust pieces of evidence in psychiatry is that of the association between mania and the first few weeks after childbirth, with a 23 times increased risk of first-onset mania in the 30 days postpartum compared with 12 months after giving birth^20^. Although the effect we found for perimenopause is much smaller than that observed for childbirth, our results support the theory that there is a link between mania/bipolar disorder and reproductive events beyond childbirth. It has been suggested that menopausal mood disorders could be predicted by sensitivity to estradiol, with 27% of individuals sensitive to either withdrawal (7%) or absolute change (20%) in estradiol levels^21^. As the postpartum period is associated with a sharp decline in estradiol levels, it may be that individuals with an estradiol-sensitive predisposition may have already developed an onset of mania during postpartum, reducing the number of first onsets at perimenopause.

Contrary to mania, we did not find any specific association between schizophrenia spectrum disorders and the perimenopause. While the lack of effect may be due to the small sample size, we did find that the incidence rate of schizophrenia spectrum disorder was significantly lower in the postmenopause compared with the premenopausal stage. Our findings, therefore, do not support the widely discussed hypothesis that hypoestrogenism may trigger the first onset of schizophrenia but are in line with those of a large collaborative study on over 130,000 incident cases of schizophrenia, showing a decline in new onsets in women after the age of 40 years^22^.

Our results corroborate findings from previous studies that have observed an increased risk of depression in the perimenopause^3,4,8–10^. The effect was smaller than that for mania, but the CIs were narrower, as there were more people who developed major depression than people who developed mania. Interestingly, while rates of first-onset MDD decreased in the postmenopause, rates of first-onset depressive symptoms remained high in the postmenopause (Supplementary Table 4). The mechanisms underpinning the link between first-onset depressive symptoms and reproductive aging may, therefore, be complex and include not only hormonal changes associated with the perimenopause, but also biopsychosocial challenges associated with aging. Depression is likely an umbrella term for several conditions with heterogeneous disease pathways. It is then possible that onsets at different reproductive stages are linked to different mechanisms. Given that our study ascertained age at first diagnosis by a health care professional, it is also possible that some participants who experienced depressive symptoms during the perimenopause delayed seeking help or that their symptoms had become severe enough to seek help only in the postmenopause. Curiously, although the male analysis revealed a similar trend of depression risk, upon closer inspection of the number of onsets around the FMP proxy (Fig. 2), they lack the sharp peak present in the female analysis. The present study observed a decline in risk of a first onset of MDD during the postmenopausal period compared with premenopause in both the female and male proxy analysis. Although older populations often have high prevalence of depression^23^, with the course of MDD becoming poorer with age^24^, our results are in line with previous studies which observe a decline in first-ever onsets of depression in the decade following the average age at FMP (~50 years of age)^25–28^. As such, it may be that while the onset of depression occurs during perimenopause or earlier in life, some individuals may remain depressed during the postmenopause, leading to a high prevalence.

### Strengths and limitations

Using data from the UK Biobank has allowed us to use a reported age at menopause to determine reproductive timing and, thus, estimate time periods to capture the pre-, peri- and postmenopausal life stages. This is a major strength over many previous studies, which have often used chronological age as a proxy for menopause despite the large variability in reproductive timing between individuals^14^. However, the UK Biobank does not include additional information on the menstrual cycle, which would provide more fine grain information on the stages of reproductive aging and menopausal symptoms. Aware of this UK Biobank limitation and of the variability in the length of the early menopause transition, we focused our analyses on the late menopausal transition and the early postmenopause, as: (1) they have less variability in their length^29^ and (2) previous studies have suggested that the late menopause transition is the period of highest risk of new-onset depression^9^. We then chose the reference period of 6–10 years before the FMP, as only very few people will experience a late menopausal transition longer than 6 years^29^.

Another unique strength of the UK Biobank is that the large sample size, the collection on information on FMP and the cohort design allow inference on a range of psychiatric disorders, including less prevalent and more severe ones, such as mania and psychosis. However, due to the particularly low prevalence of schizophrenia spectrum disorders, we were not able to run fine-grained analyses considering the different disorders separately. Further replication of our results should be carried out before definitive conclusions can be drawn. Additionally, representativeness of UK Biobank has been an area of controversy, with diverging views on the validity of its measures of association^30,31^. Our sensitivity analyses show the robustness of our findings across several socioeconomic, health and lifestyle characteristics. The increased incidence of psychiatric conditions at perimenopause were present across different levels of the Townsend Deprivation Index, BMI categories, alcohol intake frequencies and in previous and never smokers. Additionally, we note that our study design selects for those without a diagnosis of severe mental illness before 10 years before the FMP and, thus, will be ‘healthier’ by definition as is the design of the study, rather than due to an unintended selection bias.

We emphasize that the incidence and prevalence estimates of our study are not the focus of the paper and should not be taken as representative of the UK population as a whole. Moreover, our study focused on the effect of the perimenopause on the first incidence of psychiatric disorders. By using as a reference group people 10 years before their FMP, by design, we selected a population that has reached the middle age without any psychiatric diagnosis. This study design has the advantage of excluding the well-established peak of severe psychiatric disorders in early adulthood, which would mask the effect of the perimenopause.

### Directions for future work

Our study focused on the first onset of psychiatric disorders during the perimenopause. The study of recurrence of preexisting psychiatric disorders was beyond the scope of this paper and would have required a different study design. Further research focusing on large cohorts of people with previous history of mental illness is necessary to improve their risk prediction and mitigation associated with reproductive aging.

## Conclusions

Our results highlight the importance of diagnostic accuracy in the assessment of psychiatric phenomena associated with aging. First, research and clinicians should consider interpersonal variability in reproductive aging rather than use demographic age as a proxy. Second, differential diagnosis is key, and not all psychiatric symptoms with onset at the perimenopause should be considered depressive symptoms etiologically related to the perimenopause. Mechanistic research needs to differentiate between clinical manifestation that are mostly epiphenomena from those that may be triggered by the physiological changes associated with reproductive aging. Clinically, the link with mania was particularly striking and significant: although first-onset bipolar disorder is usually associated with younger age, the perimenopause represents a period of increased risk of manic onset in those without previous psychiatric history of mania. Primary care physicians should be aware of this late onset presentation of bipolar disorder to avoid the risks associated with a delayed diagnosis and misdiagnosis with depression. Antidepressants without mood stabilizers can in fact precipitate manic episodes in those with bipolar diathesis^32^.

## Methods

### Study design and participants

The initial sample consisted of 502,357 individuals from the UK Biobank^17^. The UK Biobank is a large-scale biomedical database designed to enable research into genetic and environmental determinants of disease. Participants were recruited at 40–69 years of age between 2006 and 2010 and undertook extensive data collection. Measurements included assessment center visits, genotyping and longitudinal follow-up of health outcomes including linkage to medical records. The North West Multi-Centre Ethics Committee granted ethical approval to the UK Biobank, and all participants provided written informed consent. This study was conducted under project number 13310.

Psychiatric diagnoses were assessed using interviews and a self-report web-based questionnaire (the UK Biobank mental health questionnaire). Interviews were conducted by trained nurses and included questions on past and current medical conditions and date of diagnosis. The mental health questionnaire was based on the World Health Organization’s Composite International Diagnostic Interview Short Form^33^ and integrated elements of other validated tools, such as the Patient Health Questionnaire (9-question version), the Generalized Anxiety Disorder Questionnaire 7-item^34^ and bespoke additional questions. Although some participants also have linked medical primary health care records and hospital admission records, these data were not used in the present study as only more recent records are available electronically; thus, admission dates were not trusted as valid proxies for first onsets of psychiatric disorders.

Sample demographic variables include ethnicity. This was self-reported via touchscreen questionnaire at the initial assessment center visit by the question: ‘What is your ethnic background?’.

### Selection of individuals for analysis

Figure 1 illustrates sample selection. The UK Biobank dataset was restricted to participants of female sex, as reported in the NHS central registry at the time of recruitment; although, in some instances this was updated by the participant (field identification (ID) 31). Of the 502,357 participants in the UK Biobank, 273,293 (54.4%) were of female sex. The sample was then limited to the 170,556 participants (62.4%) that had reported menopause occurrence (field ID 2724), which is defined as the cessation of menstrual periods for 12 months^29^. At each assessment center visit, participants were asked to self-report if menopause had occurred via touchscreen questionnaire (field ID 2724). Of the 22,971 individuals (13.5%) who had answered this question at multiple assessment center visits, 42 individuals (0.18%) who answered ‘yes’ but then responded ‘no’ to the same question at a subsequent visit were excluded, leaving 170,514 participants in the sample. Age at menopause, defined as age at the FMP, was asked via touchscreen questionnaire (field ID 3581) if the participant had responded ‘yes’ when asked if menopause had occurred. As this question was administered at each assessment center visit, 13,311 participants (7.81%) answered the question more than once. Of these, 442 participants (3.32%) reported different ages when asked again, and 330 (2.48%) whose reported ages at menopause varied by two or more years were excluded from the study due to potential unreliability. For the 112 individuals (0.84%) with multiple reported ages at menopause that did not vary by two or more years, the response from the earliest available assessment center visit was taken to reduce the probability of recall bias. A total of 10,726 participants (6.30%) had no age at menopause reported, having responded, ‘do not know’ or ‘prefer not to answer’ and were subsequently excluded from the study. A further 6,487 (4.07%) reported an age at menopause (field ID 3581) before 40 years of age and were excluded, as primary ovarian insufficiency is associated with major depression^35^, leaving 152,971 participants in the sample. A total of 15,490 participants (10.1%) who reported a hysterectomy (field ID 3591) or a bilateral oophorectomy (field ID 2834) were excluded from the study as these procedures have also been linked with an increased risk of depression^36–38^. Those who had undergone uterine ablations (8,988) were excluded, as this can cause cessation of menstrual periods (field ID 41272, OPCS4 codes: Q07, Q08, Q10, Q16). As contraceptive use can also cause cessation of menstrual periods, 199 participants (0.15%) who reported using a hormonal intrauterine device (IUD) or who reported starting oral contraceptives at the same age they experienced menopause were excluded from analysis, resulting in a sample size of 128,294.

### Diagnostic criteria

First onsets of MDD, mania, schizophrenia spectrum disorders and an ‘other diagnoses’ category were investigated in the present study. These disorders were chosen on the basis of previous literature investigating the effect of childbirth^20^, as these events both cause changes in sex hormone levels, and thus, new onsets of disorders during these periods may share hormone-driven etiological pathways^2,21^.

The diagnosis of MDD was based on answers from the online mental health questionnaire and mapped on the Diagnostic and Statistical Manual of Mental Disorders, Fifth Edition, (DSM-5) criteria. Such approach has been previously validated by Cai and colleagues^39^, who found this approach to have higher SNP-based heritability than any other definition of depression available in the UK Biobank, including medical record-based criteria. Details of this diagnostic criteria are available in Supplementary Note: Diagnostic Criteria. Briefly, a classification of MDD required at least two cardinal symptoms, as well as at least five total symptoms, and excluded individuals with a history of substance abuse and/or manic/psychotic conditions. Age at first onset was defined as the age at first episode of depression (field ID 20433). Analyses of individuals self-reporting depressive symptoms are available in Supplementary Note: Diagnostic Criteria (Supplementary Table 4).

The diagnoses of mania/bipolar/manic–depression (henceforth referred to as ‘mania’), schizophrenia spectrum disorders and an ‘other diagnoses’ category were based on the nurse-conducted interviews (field IDs 20002 and 20009). Individuals who reported having schizophrenia at interview were combined with individuals who reported a diagnosis of schizophrenia or ‘any other type of psychosis or psychotic illness’ in those who completed the mental health questionnaire (field IDs 20544 and 20461) to form a ‘schizophrenia spectrum disorders’ group. The ‘other diagnoses’ group included participants who reported any of the following conditions at a nurse-conducted interview: ‘anxiety/panic attacks’, ‘substance abuse/dependency’, ‘post-traumatic stress disorder’, anorexia/bulimia/other eating disorder’, ‘stress’, ‘obsessive compulsive disorder’ or ‘insomnia’.

Finally, a combined ‘psychiatric disorder’ group was formed of all the above diagnoses combined. In the case of the combined ‘psychiatric disorder’ group, if multiple diagnoses were present in a single participant, the earliest age at onset was prioritized.

For each diagnosis group, individuals were removed from analysis if they met the diagnostic criteria but did not have onset age data available, as detailed in Supplementary Note: Diagnostic Criteria.

### Statistical analysis

Age at first onset of any given psychiatric disorder relative to age at FMP was calculated as self-reported age at menopause (field ID 3581) subtracted from the age at onset. These values were grouped to form the following three life stages: premenopause (6–10 years before the FMP), perimenopause (2 years before and 2 years following FMP) and postmenopause (6–10 years after the FMP). The values between 2 and 6 years from the FMP were excluded to increase distinction between the time periods and to minimize the likelihood of misclassification due to inaccuracies in menopausal timing. The premenopause represents the reference life stage against which the other life stages were compared. The late reproductive stage (6–10 years before the FMP) was used as the refence premenopausal period to reduce the effect of recall bias and to minimize inclusion of adolescent and postnatal onsets within the reference period.

A Kaplan–Meier survival analysis was used for each diagnosis group to determine the likelihood of disorder onset at each life stage. This was conducted using the ‘survival’ R package in RStudio, version 4.2.1 (refs. ^40,41^). Incidence rates in person-years for each time period were calculated as the number of cases divided by four times the total number of cases and controls, as each time period was 4 years long. Incidence RRs were calculated to compare the rate of first onsets in the perimenopause and postmenopause compared with the premenopausal stage, using the two-sided ‘rateratio.test’ R package^42^, with a confidence level of 0.95. The false discovery rate correction was applied to correct for 50 tests (10 tests in Table 2, 30 tests in Supplementary Table 1, 8 tests in Supplementary Table 3 and 2 tests in Supplementary Table 4)^43^. We provide *P* values both with and without correction for multiple testing.

### Sensitivity analyses

Previous literature has observed that the UK Biobank participants differ from UK Census data on several socioeconomic, health and lifestyle characteristics^30^. Participants have been found to be more likely to live in less deprived geographical areas and less likely to be obese, to currently smoke or to drink alcohol daily. To consider the effect of this sampling bias, we investigated the effects of Townsend Deprivation Index, BMI, smoking status and alcohol intake frequency on the findings of the present study by stratifying the sample on these characteristics. Further details are available in Supplementary Note: Sensitivity Analysis.

### Analyses of male participants

It is possible that the observed effects may not be driven by the hormonal fluctuations that characterized the perimenopause. Rather, they could be driven by age or by unknown ascertainment and assessment bias. The same analyses were, therefore, conducted in male participants enrolled in UK Biobank.

First, we matched male participants to female participants on the basis of their most recent age that they came into the assessment center (field ID 21003). Then, for each male participant, we constructed a timeline centered around the age at the FMP of their matched female participant. So, for example, if we matched a female and male participant based on the age at the last assessment of 62 and the female participant had an age at the FMP of 50 years, we then centered the timeline of the male participant around 50. The three time periods considered as a proxy in this case would be 40–44 years for premenopause, 48–52 years for perimenopause and 56–60 years for postmenopause. This was carried out to account for the variation and distribution of menopausal timing in female participants to consider the effect of attrition based on the age of most recent follow-up and to create equal sample sizes to homogenize the statistical power. This resulted in 128,294 male participants being included in analysis. All following statistical analyses mirrored the analyses conducted in the female sample.

### Patient and public involvement

People with lived experience were involved in the design of the study, in the interpretation of the results and in the writing of the manuscript. C.D., a researcher with lived experience and coauthor on the publication, provided expertise and voiced the issues experienced by people affected by severe mental illness. In her roles within the charities Bipolar UK and Action on Postpartum Psychosis, C.D. has provided support to women with lived experience of illness during the perimenopause over several years and has worked as patient and public involvement lead. Moreover, the design of the current study was informed by a Bipolar UK survey designed and conducted by C.D., which received over 1,000 responses,

### Reporting summary

Further information on research design is available in the Nature Portfolio Reporting Summary linked to this article.

## Supplementary information

Supplementary Information Supplementary methods (diagnostic criteria and sensitivity analysis), Tables 1–4 and Figs. 1 and 2.

Reporting Summary

## Data Availability

All the data used in this study, both raw and derived, are available from the UK Biobank (https://www.ukbiobank.ac.uk/). This study was conducted under project number 13310. Our access to the data does not allow for data redistribution.

## Code availability

Code used for analysis is available online at https://lms-j.github.io/perimeno-first-onsets/.
