# Supplementary material for: Exploration of first onsets of mania, schizophrenia spectrum disorders and major depressive disorder in perimenopause
Source: Nat Ment Health. 2024 Aug 15;2(10):1161–8. doi: 10.1038/s44220-024-00292-4 (PMC11479941; doi:10.1038/s44220-024-00292-4)
Supplement: Supplementary file 1 — Supplementary methods (diagnostic criteria and sensitivity analysis), Tables 1–4 and Figs. 1 and 2. [file 44220_2024_292_MOESM1_ESM.pdf]

nature mental health

Supplementary information https://doi.org/10.1038/s44220-024-00292-4

Exploration of first onsets of mania, schizophrenia spectrum disorders and major depressive disorder in perimenopause

In the format provided by the authors and unedited

SUPPLEMENTARY NOTE

Diagnostic Criteria 1
Diagnostic criteria for major depressive disorder 1
Diagnostic criteria for depressive symptoms, mania, and other diagnoses 1
Diagnostic criteria for schizophrenia spectrum disorders 2
Sensitivity Analysis 3
Townsend Deprivation Index 3
Body mass index 3
Smoking status 3
Alcohol intake frequency 3
Supplementary Table 1: Stratified analysis of socioeconomic, health, and lifestyle characteristics. 5
Supplementary Table 1 (continued): Stratified analysis of socioeconomic, health, and lifestyle characteristics. 6
Supplementary Table 2: Demographic characteristics of male participants. 7
Supplementary Table 3: Incidence rates of psychiatric disorders in male participants during the matched “perimenopause” and “postmenopause” proxies compared to the “premenopause” stage proxy. 8
Supplementary Table 4: Incidence rates of depressive symptoms during the perimenopause and postmenopause compared to the premenopause stage. 9
Supplementary Figure 1: Distribution of the age at final menstrual period (FMP). 10
Supplementary Figure 2: Incidence and prevalence of female participants. 11
Supplementary References 13

Diagnostic Criteria

Diagnostic criteria for major depressive disorder

Major depressive disorder (MDD) was akin to the 'strictly defined major depressive disorder' phenotype previously described by Cai et al.[1] This phenotype is based on answers from a mental health questionnaire which was completed online and is based on the DSM-V criteria for MDD.

To classify as having MDD, individuals were required to have at least two cardinal symptoms (field IDs 20441 and 20446), and at least 5 total symptoms in total, including cardinal symptoms (field IDs: 20435, 20437, 20440, 20449, 20450, 20532, 20536). Details of accepted answers have been described previously.[1] As per the Cai et al criteria, we excluded individuals with a history of substance abuse (field ID 20002 codes 1408-1410) and/or manic/psychotic conditions (field ID 20002 codes 1289 and 1291; field ID 20126 codes 1 and 2). Age at first onset was defined as the age at first episode of depression (field ID 20433).

Of 128 294 individuals in the sample, 43 856 answered the mental health questionnaire. Of these, 6585 met the criteria for strictly defined lifetime MDD as outlined by Cai et al. Of the 6585 who met the criteria, 6225 had an onset age from the mental health questionnaire question: "About how old were you the FIRST time you had a period of two weeks like this? (Whether or not you received any help for it)" (field ID 20433).

Diagnostic criteria for depressive symptoms, mania, and other diagnoses

The following section details the diagnostic criteria for depressive symptoms, mania, and other diagnoses. Results for depressive symptoms are not in the main manuscript and are instead included in **Supplementary Table 4**.

Depressive symptoms, mania, and other diagnoses were determined by verbal interviews held by a trained nurse at assessment centre visits. Participants were asked "Has a doctor ever told you that you have any other serious medical conditions?", where "any other" refers to any non-cancer conditions (field ID 20002). For each medical condition reported, participants were then asked when this condition was first diagnosed by a doctor. This was either given as an age in years or, if a calendar year was reported, then their age at the mid-point of that year was used (field ID 20009).

8525 participants reported a diagnosis of depressive symptoms ('depression': illness code 1286). Of these, 134 had no corresponding onset age, leaving a total of 8391 participants with depressive symptoms.

355 participants had a diagnosis of mania ('mania/bipolar disorder/manic depression': illness code 1291). Of these, 3 participants did not have a corresponding age at onset available and thus were excluded, leaving 352 individuals with mania in the study.

3011 participants reported a diagnosis that fell into the 'other diagnosis' category. This included reporting having a diagnosis for any of the following: 'anxiety/panic attacks': illness code 1287; 'substance abuse/dependency': illness codes 1408-1410; 'post-traumatic stress disorder': illness code 1469; 'anorexia/bulimia/other eating disorder':

illness code 1470; ‘stress’: illness code 1614; ‘obsessive compulsive disorder’: illness code 1615; ‘insomnia’: illness code 1616. Of these 3011 participants, 56 had no corresponding onset age, leaving a total of 2955 participants in the ‘other diagnoses’ group.

Diagnostic criteria for schizophrenia spectrum disorders

Diagnosis sources for schizophrenia spectrum disorders included both the nurse-administered verbal interview and the mental health questionnaire. From the verbal interview, participants were classified as having a schizophrenia spectrum disorder if they reported a diagnosis of schizophrenia (field ID 20002, illness code 1289). From the mental health questionnaire, a schizophrenia spectrum disorders diagnosis was determined by answers of ‘Schizophrenia’ and/or ‘Any other type of schizophrenia spectrum disorders or psychotic illness’ to the question “Have you been diagnosed with one or more of the following mental health problems by a professional, even if you don't have it currently? (tick all that apply):” (field ID 20544).

Age at onset was obtained from the onset age corresponding to a diagnosis of schizophrenia from the nurse-administered interview (field ID 20009). If this was not available, then the answer to the following question from the mental health questionnaire was used: “How old were you (approximately) when you first had one of these experiences (seeing a vision, hearing a voice, or believing that something strange was trying to communicate with you, or there was a plot against you)?” (field ID 20461).

91 individuals reported a diagnosis of schizophrenia from the nurse-administered interview. 16 individuals reported having schizophrenia from the mental health questionnaire and 91 reported ‘Any other type of schizophrenia spectrum disorders or psychotic illness’. Of these individuals, 8 reported having schizophrenia in both the interview and mental health questionnaire, whilst 1 individual reported as having both schizophrenia and ‘Any other type of schizophrenia spectrum disorders or psychotic illness’ in the mental health questionnaire. This gave a total of 189 individuals with a diagnosis.

An onset age from the interview was available for 89 individuals, with 93 individuals having an onset age from the mental health questionnaire. Of these 93, 7 also had an onset age from the interview and thus this was used. In total, 14 participants were excluded for having a schizophrenia spectrum disorders diagnosis and no corresponding onset age, leaving 175 participants with schizophrenia spectrum disorder in the study.

Sensitivity Analysis

The following sensitivity analysis was conducted on the combined ‘psychiatric disorder’ group, whereby the first onset age of any psychiatric disorder that is recorded for a participant is used. All the following characteristics were obtained either at recruitment or at the first assessment centre visit, and were chosen as they are all variables which were found to be different in UK Biobank participants compared to UK Census Data by Fry et al.[2] The sample investigated in the sensitivity analyses include only participants that met all inclusion criteria for the study, as detailed in **Figure 1** of the main manuscript (n=128 294).

Townsend Deprivation Index

Townsend Deprivation Index is a measure of material deprivation that can be calculated for populations within geographical areas based on the following variables: unemployment, non-car ownership, non-home ownership, and overcrowding.[3] A higher Townsend Deprivation Index score indicates a higher level of deprivation. Townsend Deprivation Index values were calculated for UK Biobank participants before to the participant’s enrolment in the study, based on their postcode and prior National Census Output Area data (field ID 22189). To investigate the effect of differences in Townsend Deprivation Index (very deprived neighbourhoods compared to the least deprived), incidence rate ratios were calculated for the top and bottom 10% of Deprivation Indexes within the sample.

Body mass index

Body mass index (BMI) was calculated from height and weight measurements collected at the initial Assessment Centre visit (field ID 21001). Incidence rate ratios were then calculated for the following BMI categories: underweight (<18.5), healthy (≥18.5 & <25), pre-obese (≥25 & <30), and obese (≥30).[4]

Smoking status

Current smoking status (field ID 20116) is a variable that has been derived by UK Biobank from the following two questions asked via by touchscreen questionnaire at the initial assessment centre visit: “Do you smoke tobacco now?”, (field ID 1239) and “In the past, how often have you smoked tobacco?”, (field ID 1249). From these, participants have been assigned one of the following smoking statuses: never smoker, previous smoker, and current smoker. Incidence rate ratios were calculated for each of these categories. 426 participants (0.33%) had either selected ‘Prefer not to answer’ or had a missing value and were not included in the analysis.

Alcohol intake frequency

Alcohol intake frequency was determined from response to the question, “About how often do you drink alcohol?”, asked via touchscreen questionnaire at the initial assessment centre visit (field ID 1558). Available responses were as follows: ‘Daily or almost daily’, ‘Three or four times a week’, ‘Once or twice a week’, One to three times a month’, Special occasions only’, ‘Never’, and ‘Prefer not to answer’. 78 individuals (0.06%) either answered ‘Prefer not to answer’ or had a missing value, and thus were

excluded from the analysis. Each available response to the question (excluding ‘Prefer not to answer’) formed a group for which incidence rate ratios were calculated.

Supplementary Table 1: Stratified analysis of socioeconomic, health, and lifestyle characteristics.

Characteristic	Life stage	N	New onsets	Rate per 1000 person-years	RR (95% CI)	p	Adj. p
Low Townsend Deprivation Index	Premenopause	12328	75	1.52	1.00 (Ref)		
	Perimenopause	12189	116	2.38	1.56 (1.16-2.12)*	2.86E-03*	6.23E-03*
	Postmenopause	9676	63	1.63	1.07 (0.75-1.52)	7.53E-01	8.76E-01
High Townsend Deprivaton Index	Premenopause	12254	74	1.51	1.00 (Ref)		
	Perimenopause	12136	104	2.14	1.42 (1.04-1.94)*	2.50E-02*	4.99E-02*
	Postmenopause	9050	56	1.55	1.02 (0.71-1.47)	9.57E-01	1.00E+00
Underweight BMI (<18.5)	Premenopause	981	5	1.27	1.00 (Ref)		
	Perimenopause	972	13	3.34	2.62 (0.88-9.40)	9.24E-02	1.58E-01
	Postmenopause	793	3	0.95	0.74 (0.12-3.82)	9.68E-01	1.00E+00
Healthy BMI (≥18.5 & <25)	Premenopause	48447	301	1.55	1.00 (Ref)		
	Perimenopause	47923	431	2.25	1.45 (1.25-1.68)*	8.36E-07*	4.65E-06*
	Postmenopause	37820	255	1.69	1.09 (0.91-1.29)	3.58E-01	4.98E-01
Pre-obese BMI (≥25 & <30)	Premenopause	46334	260	1.40	1.00 (Ref)		
	Perimenopause	45861	439	2.39	1.71 (1.46-2.00)*	5.06E-12*	4.21E-11*
	Postmenopause	36396	244	1.68	1.19 (1.00-1.43)	5.12E-02	9.46E-02
Obese BMI (≥30)	Premenopause	26818	186	1.73	1.00 (Ref)		
	Perimenopause	26470	247	2.33	1.35 (1.11-1.64)*	2.50E-03*	6.08E-03*
	Postmenopause	20261	133	1.64	0.95 (0.75-1.19)	6.70E-01	8.06E-01
Never smokers	Premenopause	72219	390	1.35	1.00 (Ref)		
	Perimenopause	71458	648	2.27	1.68 (1.48-1.91)*	2.66E-16*	2.66E-15*
	Postmenopause	55745	341	1.53	1.13 (0.98-1.31)	1.00E-01	1.62E-01
Previous smokers	Premenopause	40623	269	1.66	1.00 (Ref)		
	Perimenopause	40172	384	2.39	1.44 (1.23-1.69)*	3.94E-06*	1.97E-05*
	Postmenopause	32533	216	1.66	1.00 (0.83-1.20)	1.00E+00	1.00E+00
Current smokers	Premenopause	9860	93	2.36	1.00 (Ref)		
	Perimenopause	9718	99	2.55	1.08 (0.81-1.45)	6.45E-01	8.06E-01
	Postmenopause	7063	76	2.69	1.14 (0.83-1.56)	4.38E-01	5.91E-01

* Significant at α = 0.05 confidence level.; RR = Rate Ratio; CI = Confidence Interval; Premenopause = 6-10 years before FMP; Perimenopause = Between -2 years before and 2 years after FMP; Postmenopause = 6-10 years after FMP.

Adjusted p-values (Adj. p) represent the False Discovery Rate (FDR)-adjusted p-values, accounting for all tests conducted in Table 2 and Supplementary Tables 1, 3, and 4.

Table continues on following page.

Supplementary Table 1 (continued): Stratified analysis of socioeconomic, health, and lifestyle characteristics.

Characteristic	Life stage	N	New onsets	Rate per 1000 person-years	RR (95% CI)	p	Adj. p
Alcohol intake: daily	Premenopause	21999	139	1.58	1.00 (Ref)		
	Perimenopause	21737	209	2.40	1.52 (1.22-1.90)*	1.33E-04*	4.14E-04*
	Postmenopause	17705	110	1.55	0.98 (0.76-1.27)	9.47E-01	1.00E+00
Alochol intake: 3-4 times/week	Premenopause	25883	159	1.54	1.00 (Ref)		
	Perimenopause	25602	227	2.22	1.44 (1.17-1.78)*	4.23E-04*	1.24E-03*
	Postmenopause	20112	115	1.43	0.93 (0.73-1.19)	6.01E-01	7.91E-01
Alochol intake: 1-2 times/week	Premenopause	30984	180	1.45	1.00 (Ref)		
	Perimenopause	30673	261	2.13	1.46 (1.21-1.78)*	8.67E-05*	2.89E-04*
	Postmenopause	23663	155	1.64	1.13 (0.90-1.41)	2.98E-01	4.38E-01
Alochol intake: 1-3 times/month	Premenopause	15052	112	1.86	1.00 (Ref)		
	Perimenopause	14861	161	2.71	1.46 (1.14-1.87)*	2.55E-03*	6.08E-03*
	Postmenopause	11435	88	1.92	1.03 (0.77-1.38)	8.67E-01	9.85E-01
Alcohol intake: Special occasions	Premenopause	17900	93	1.30	1.00 (Ref)		
	Perimenopause	17726	160	2.26	1.74 (1.34-2.27)*	2.14E-05*	8.24E-05*
	Postmenopause	13966	111	1.99	1.53 (1.15-2.04)*	3.04E-03*	6.34E-03*
Alochol intake: Never	Premenopause	11227	68	1.51	1.00 (Ref)		
	Perimenopause	11092	113	2.55	1.68 (1.23-2.31)*	7.61E-04*	2.11E-03*
	Postmenopause	8753	58	1.66	1.09 (0.76-1.58)	6.77E-01	8.06E-01

* Significant at α = 0.05 confidence level.; RR = Rate Ratio; CI = Confidence Interval; Premenopause = 6-10 years before FMP; Perimenopause = Between -2 years before and 2 years after FMP; Postmenopause = 6-10 years after FMP.

Adjusted p-values (Adj. p) represent the False Discovery Rate (FDR)-adjusted p-values, accounting for all tests conducted in Table 2 and Supplementary Tables 1, 3, and 4.

Supplementary Table 2: Demographic characteristics of male participants.

Demographic variable	N	%	Mean	SD
Age at first assessment (years)			59.6	5.69
Age at most recent follow-up (years)			62.4	6.37
Ethnicity				
White[a]	122205	95.25		
Black or black British[b]	1358	1.06		
Mixed[c]	427	0.33		
Indian	1485	1.16		
Pakistani	441	0.34		
Bangladeshi	41	0.03		
Chinese	264	0.21		
Other Asian background[d]	422	0.33		
Other ethnic group[d]	823	0.64		
Don't know/Prefer not to say	828	0.65		

Ethnic groups have been organised to reflect the same categories as Fry et al.,[2] who in turn used these categories to allow for comparison to the 2001 and 2011 UK Census Data.

[a] Includes white British, white Irish, and any other white background.

[b] Includes black Caribbean, black African, and any other black background.

[c] Includes white and black Caribbean, white and black African, white and Asian, and any other mixed background.

[d] 'Other Asian background' and 'Other ethnic group' are both specific responses available from the question posed by UK Biobank and are not further broken down into more granular categories.

Supplementary Table 3: Incidence rates of psychiatric disorders in male participants during the matched “perimenopause” and “postmenopause” proxies compared to the “premenopause” stage proxy.

This table displays the incidence rate ratios associated with each psychiatric disorder calculated from comparisons between different life stages. The first column lists psychiatric disorders. Incidence rates are calculated as the number of first onsets per 1000 person-years. Incidence rate ratios are calculated as the ratio associated with each life stage, relative to the reference premenopause stage.

Disorder	Life stage (proxies)	N	New onsets	Rate per 1000 person-years	RR (95%CI)	p	Adj. p
Major depressive disorder	Premenopause	37719	461	3.06	1.00 (Ref)		
	Perimenopause	36925	512	3.47	1.13 (1.00-1.29)	5.30E-02	9.46E-02
	Postmenopause	34917	311	2.23	0.73 (0.63-0.84)*	1.63E-05*	6.79E-05*
Mania	Premenopause	128131	42	0.08	1.00 (Ref)		
	Perimenopause	128050	37	0.07	0.88 (0.55-1.41)	6.55E-01	8.06E-01
	Postmenopause	90323	18	0.05	0.61 (0.33-1.08)	9.46E-02	1.58E-01
Schizophrenia spectrum disorders	Premenopause	128108	21	0.04	1.00 (Ref)		
	Perimenopause	128068	12	0.02	0.57 (0.26-1.22)	1.63E-01	2.47E-01
	Postmenopause	100808	11	0.03	0.67 (0.29-1.44)	3.57E-01	4.98E-01
Other diagnoses	Premenopause	127449	201	0.39	1.00 (Ref)		
	Perimenopause	126994	289	0.57	1.44 (1.20-1.74)*	6.91E-05*	2.47E-04*
	Postmenopause	89408	192	0.54	1.36 (1.11-1.67)*	2.69E-03*	6.11E-03*

* Significant at α = 0.05 confidence level.; RR = Rate Ratio; CI = Confidence Interval; Premenopause = 6-10 years before matched “FMP”; Perimenopause = Between -2 years before and 2 years after matched “FMP”; Postmenopause = 6-10 years after matched “FMP”.

Adjusted p-values (Adj. p) represent the False Discovery Rate (FDR)-adjusted p-values, accounting for all tests conducted in Table 2 and Supplementary Tables 1, 3, and 4.

Note that age at FMP was based on values from matched female participants, as detailed under ‘Analyses of male participants’ in the methods section of the main manuscript.

Supplementary Table 4: Incidence rates of depressive symptoms during the perimenopause and postmenopause compared to the premenopause stage.

This table displays the incidence rate ratios associated with each psychiatric disorder calculated from comparisons between different life stages. The first column lists psychiatric disorders. Incidence rates are calculated as the number of first onsets per 1000 person-years. Incidence rate ratios are calculated as the ratio associated with each life stage, relative to the reference premenopause stage.

Disorder	Life stage	N	New onsets	Rate per 1000 person-years	RR (95% CI)	p	Adj. p
Depressive symptoms	Premenopause	125212	718	1.43	1.00 (Ref)		
	Perimenopause	123607	1346	2.72	1.90 (1.73-2.08)*	9.01E-46*	4.51E-44*
	Postmenopause	86292	659	1.91	1.33 (1.20-1.48)*	1.40E-07*	8.72E-07*

* Significant at $\alpha = 0.05$ confidence level.; RR = Rate Ratio; CI = Confidence Interval; Premenopause = 6-10 years before FMP; Perimenopause = Between -2 years before and 2 years after FMP; Postmenopause = 6-10 years after FMP.

Adjusted p-values (Adj. p) represent the False Discovery Rate (FDR)-adjusted p-values, accounting for all tests conducted in Table 2 and Supplementary Tables 1, 3, and 4.

Supplementary Figure 1: Distribution of the age at final menstrual period (FMP).

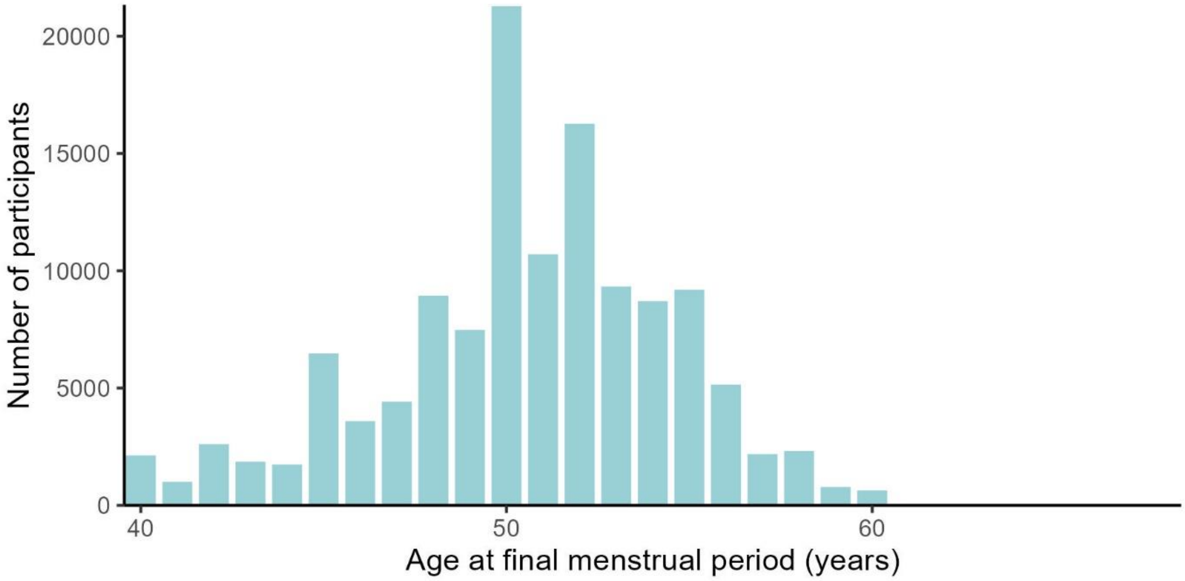

Supplementary Figure 2: Incidence and prevalence of female participants.

Note that prevalence is lower than in the total UK Biobank sample, as those with no reported onset age are excluded. Additionally, not all participants have reached 80 years-of-age, and thus final prevalences are likely to be underestimated.

A. Major Depressive Disorder

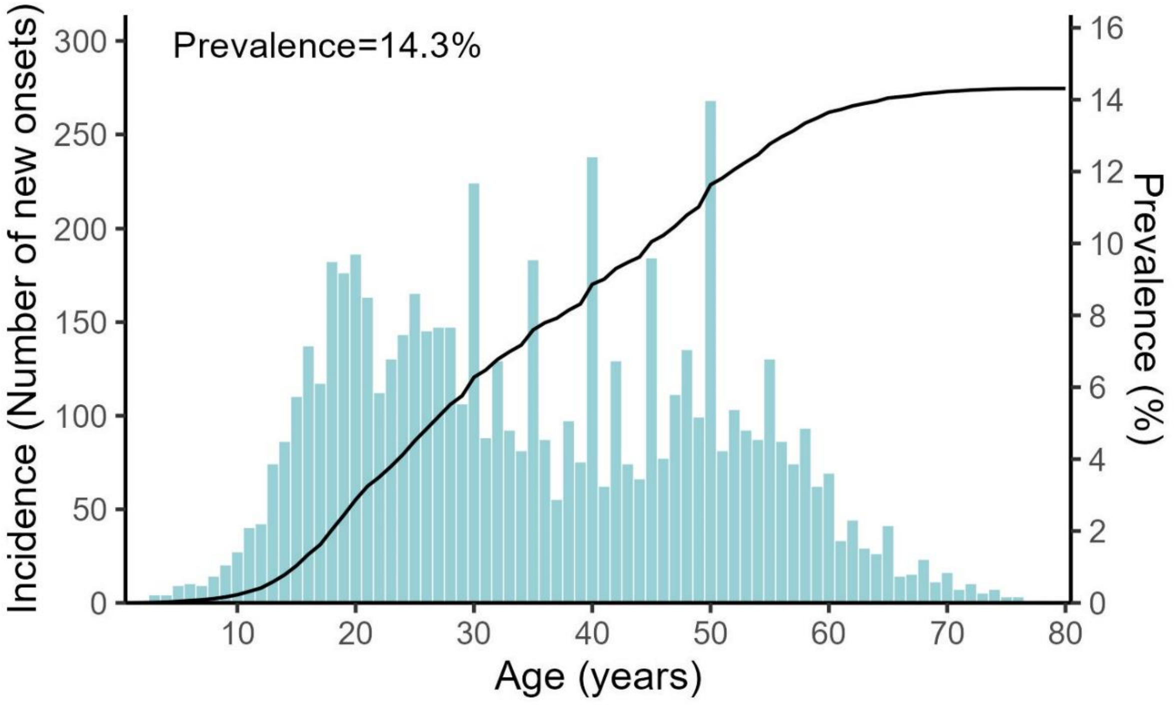

B. Mania

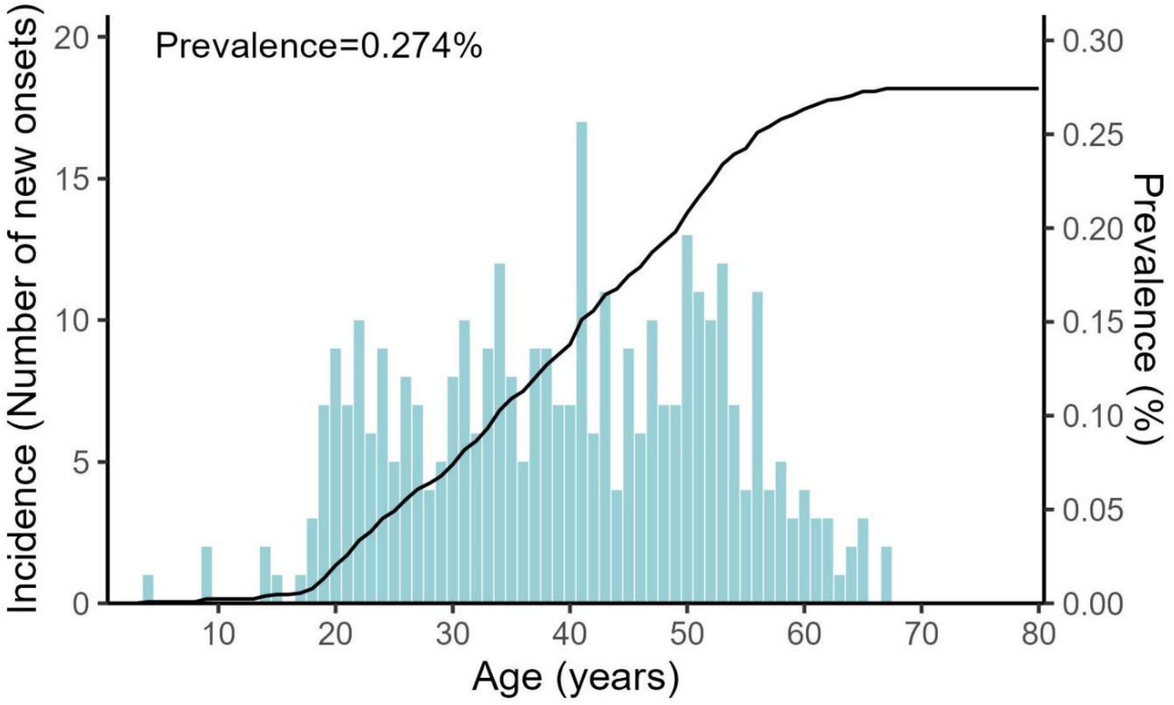

C. Schizophrenia spectrum disorders

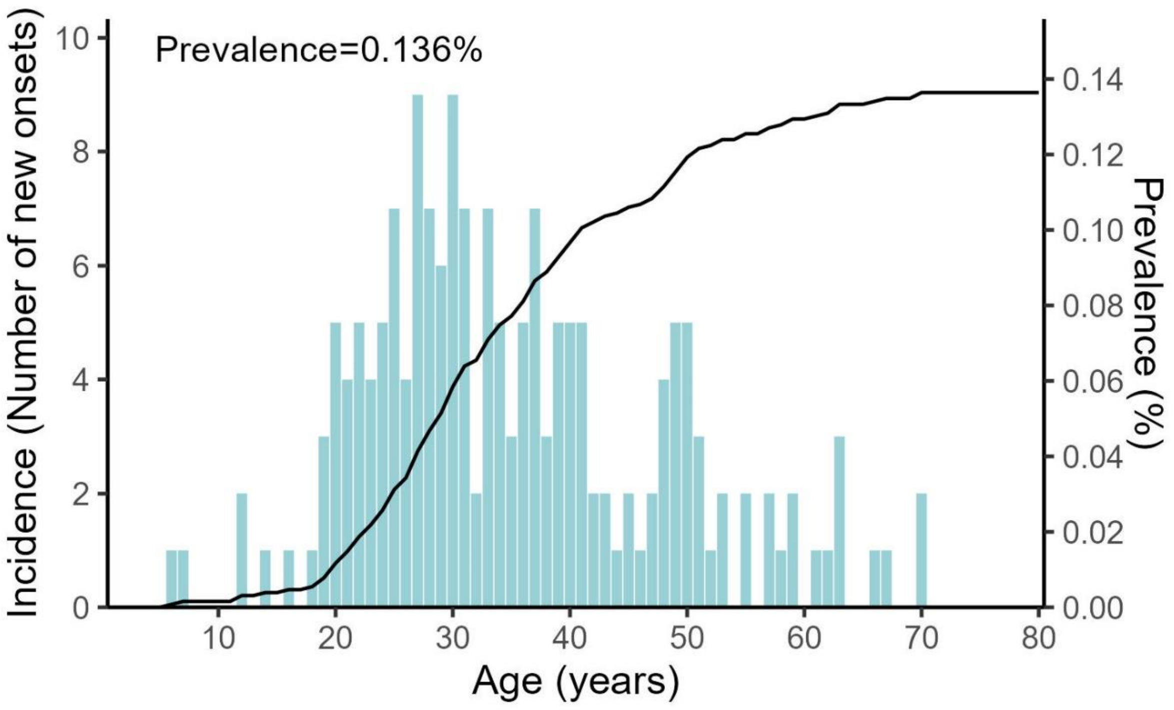

D. Other diagnoses

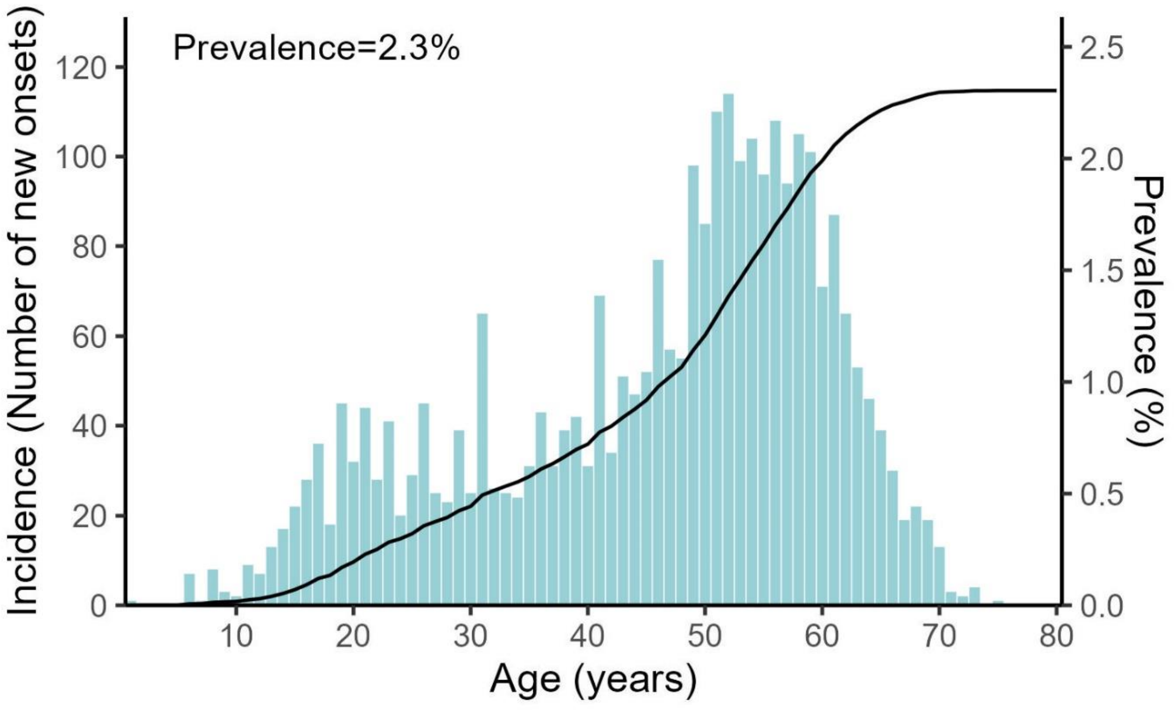

Supplementary References

1 Cai N, Revez JA, Adams MJ, *et al.* Minimal phenotyping yields genome-wide association signals of low specificity for major depression. *Nat Genet* 2020; **52**: 437–47.

2 Fry A, Littlejohns TJ, Sudlow C, *et al.* Comparison of Sociodemographic and Health-Related Characteristics of UK Biobank Participants With Those of the General Population. *Am J Epidemiol* 2017; **186**: 1026–34.

3 Townsend P, Phillimore P, Beattie A. Health and Deprivation: Inequality and the North. London: Routledge, 1988 DOI:10.4324/9781003368885.

4 The World Health Organization. A healthy lifestyle - WHO recommendations. 2010; published online May 6. https://www.who.int/europe/news-room/fact-sheets/item/a-healthy-lifestyle---who-recommendations (accessed March 19, 2024).
