# Supplementary material for: Exploration of first onsets of mania, schizophrenia spectrum disorders and major depressive disorder in perimenopause
Source: Nat Ment Health. 2024 Aug 15;2(10):1161–8. doi: 10.1038/s44220-024-00292-4 (PMC11479941; doi:10.1038/s44220-024-00292-4)
Supplement: Supplementary file 2 — Reporting Summary [file 44220_2024_292_MOESM2_ESM.pdf]

nature portfolio

Corresponding author(s): Arianna Di Florio

Last updated by author(s): Jun 4, 2024

Reporting Summary

Nature Portfolio wishes to improve the reproducibility of the work that we publish. This form provides structure for consistency and transparency in reporting. For further information on Nature Portfolio policies, see our Editorial Policies and the Editorial Policy Checklist.

Statistics

For all statistical analyses, confirm that the following items are present in the figure legend, table legend, main text, or Methods section.

n/a	Confirmed	
☐	☒	The exact sample size (*n*) for each experimental group/condition, given as a discrete number and unit of measurement
☐	☒	A statement on whether measurements were taken from distinct samples or whether the same sample was measured repeatedly
☐	☒	The statistical test(s) used AND whether they are one- or two-sided<br>*Only common tests should be described solely by name; describe more complex techniques in the Methods section.*
☐	☒	A description of all covariates tested
☒	☐	A description of any assumptions or corrections, such as tests of normality and adjustment for multiple comparisons
☐	☒	A full description of the statistical parameters including central tendency (e.g. means) or other basic estimates (e.g. regression coefficient) AND variation (e.g. standard deviation) or associated estimates of uncertainty (e.g. confidence intervals)
☐	☒	For null hypothesis testing, the test statistic (e.g. *F*, *t*, *r*) with confidence intervals, effect sizes, degrees of freedom and *P* value noted<br>*Give P values as exact values whenever suitable.*
☒	☐	For Bayesian analysis, information on the choice of priors and Markov chain Monte Carlo settings
☒	☐	For hierarchical and complex designs, identification of the appropriate level for tests and full reporting of outcomes
☒	☐	Estimates of effect sizes (e.g. Cohen's *d*, Pearson's *r*), indicating how they were calculated

Our web collection on statistics for biologists contains articles on many of the points above.

Software and code

Policy information about availability of computer code

Data collection	Software was not required for data collection.
Data analysis	All data analysis was conducted in R, using RStudio, version 4.2.1. R packages used include tidyverse (version 2.0.0), survival (version 3.5-8), and rateratio.test (version 1.1), as detailed and cited in the methods section. Code used for analysis is available online at https://lms-j.github.io/perimeno-first-onsets/.

For manuscripts utilizing custom algorithms or software that are central to the research but not yet described in published literature, software must be made available to editors and reviewers. We strongly encourage code deposition in a community repository (e.g. GitHub). See the Nature Portfolio guidelines for submitting code & software for further information.

Data

Policy information about availability of data

All manuscripts must include a data availability statement. This statement should provide the following information, where applicable:

- Accession codes, unique identifiers, or web links for publicly available datasets
- A description of any restrictions on data availability
- For clinical datasets or third party data, please ensure that the statement adheres to our policy

All the data used in this study, both raw and derived, are available from the UK Biobank (https://www.ukbiobank.ac.uk/). This study was conducted under project number 13310. Our access to the data does not allow for data redistribution. We have included a data availability statement in our manuscript.

Research involving human participants, their data, or biological material

Policy information about studies with human participants or human data. See also policy information about sex, gender (identity/presentation), and sexual orientation and race, ethnicity and racism.

Reporting on sex and gender	The term 'sex' is used and defined in this manuscript, as detailed by UK Biobank (https://biobank.ctsu.ox.ac.uk/crystal/field.cgi?id=31). Sex was reported in the NHS central registry at the time of recruitment, although in some instances this was updated by the participant.
Reporting on race, ethnicity, or other socially relevant groupings	Self-reported ethnicity proportions are available in Table 1. Ethnic groups were collated to reflect those reported previously by Fry et al. 2017 (PMID: 28641372), who used groups comparable to those from UK Census Data to examine the representativeness of UK Biobank.
Population characteristics	Our sample consisted of 128 294 participants from the UK, all of whom were female and had reported occurrence of menopause. The mean age at most recent follow-up for our sample was 62.5 years (standard deviation = 6.30 years). Further information of the population characteristics are detailed in Table 1.
Recruitment	The present study used secondary data provided by UK Biobank. Participant recruitment has been previously described (PMID: 25826379).
Ethics oversight	The North West Multi-Centre Ethics Committee granted ethical approval to UK Biobank and all participants provided written informed consent. This study was conducted under project number 13310.

Note that full information on the approval of the study protocol must also be provided in the manuscript.

Field-specific reporting

Please select the one below that is the best fit for your research. If you are not sure, read the appropriate sections before making your selection.

☐ Life sciences ☒ Behavioural & social sciences ☐ Ecological, evolutionary & environmental sciences

For a reference copy of the document with all sections, see nature.com/documents/nr-reporting-summary-flat.pdf

Behavioural & social sciences study design

All studies must disclose on these points even when the disclosure is negative.

Study description	This is a cohort study involving investigations of quantitative data.
Research sample	Our sample consisted of 128 294 post-menopausal female participants from UK Biobank, who were recruited at 40-69 years-of-age between 2006-2010. This sample was chosen due to the wealth of data available in UK Biobank, which has specifically asked participants questions on first onset psychiatric illness and age at final menstrual period, in an epidemiological study large enough to detect rare outcomes such as first incidence of mania and schizophrenia spectrum disorders.<br>Previous literature by Fry et al. has investigated the representativeness of this sample (PMID: 28641372). The paper found that, although the UK Biobank sample consists of mostly those reporting white ethnicity, ethnicity proportions were similar to those from UK Census data of individuals in the same age range. As stated in the guidance outlined in Fry's paper, a sample which is ethnically similar to the UK population would not be a suitable study design for detecting the effects of ethnicity as an exposure. We have instead used the UK Biobank to investigate the associations between exposure (menopause) and disease (psychiatric disorders), as recommended.<br>Fry's paper discusses several variables which were found to differ between UK Biobank and the general population (e.g. the Townsend Deprivation Index, BMI, smoking status, alcohol consumption, etc). In light of this, we have conducted an investigation into the effect of these variables in our study, which is included in the Supplementary Note (Supplementary Table 4). The increased incidence of psychiatric conditions at perimenopause were present across different BMI categories, alcohol intake frequencies and in previous and never-smokers.
Sampling strategy	The present study used a secondary dataset, UK Biobank, for which the sample size and original sampling strategy has been previously described in detail (PMID: 25826379). From the larger UK Biobank dataset (n=502,357), data from all participants whom met the inclusion criteria were analysed in the present study (n=128,294). Inclusion criteria are fully described in Figure 1, and included the following: female sex; menopause age available; no hysterectomy/bilateral oophorectomy/uterine ablation; no hormonal IUD; no contraceptive pill use starting at the age at menopause.
Data collection	All data used in this study was collected by UK Biobank (PMID: 25826379). Data utilised in the present study were collected via touchscreen questionnaire and by nurse-conducted interviews at assessment centre visits. As the present study uses secondary data and the data was collected as part of a cohort study, researchers involved in collecting data were not required to be blinded.
Timing	Participants were recruited at 40-69 years-of-age between 03/2006 - 10/2010. All data were collected by UK Biobank (PMID: 25826379).
Data exclusions	Participants were excluded by criteria detailed in the 'Sampling strategy' section above.

Non-participation	The present study uses secondary data collected by UK Biobank (PMID: 25826379). In the original data collection by UK Biobank, 503,325 participants were recruited from the 9.2 million invitations sent out - a response rate of 5.47% (https://doi.org/10.1016/j.hlpt.2012.07.003). The representativeness of this sample has been discussed above in the 'Research sample' section.
Randomization	Randomization was not conducted due to the observational study design.

Reporting for specific materials, systems and methods

We require information from authors about some types of materials, experimental systems and methods used in many studies. Here, indicate whether each material, system or method listed is relevant to your study. If you are not sure if a list item applies to your research, read the appropriate section before selecting a response.

Materials & experimental systems

n/a	Involved in the study
☒	☐ Antibodies
☒	☐ Eukaryotic cell lines
☒	☐ Palaeontology and archaeology
☒	☐ Animals and other organisms
☒	☐ Clinical data
☒	☐ Dual use research of concern
☒	☐ Plants

Methods

n/a	Involved in the study
☒	☐ ChIP-seq
☒	☐ Flow cytometry
☒	☐ MRI-based neuroimaging

Plants

Seed stocks	*Report on the source of all seed stocks or other plant material used. If applicable, state the seed stock centre and catalogue number. If plant specimens were collected from the field, describe the collection location, date and sampling procedures.*
Novel plant genotypes	*Describe the methods by which all novel plant genotypes were produced. This includes those generated by transgenic approaches, gene editing, chemical/radiation-based mutagenesis and hybridization. For transgenic lines, describe the transformation method, the number of independent lines analyzed and the generation upon which experiments were performed. For gene-edited lines, describe the editor used, the endogenous sequence targeted for editing, the targeting guide RNA sequence (if applicable) and how the editor was applied.*
Authentication	*Describe any authentication procedures for each seed stock used or novel genotype generated. Describe any experiments used to assess the effect of a mutation and, where applicable, how potential secondary effects (e.g. second site T-DNA insertions, mosiacism, off-target gene editing) were examined.*
